# Impact of plasma histones in human sepsis and their contribution to cellular injury and inflammation

**DOI:** 10.1186/s13054-014-0543-8

**Published:** 2014-09-24

**Authors:** Michael Liembo Ekaney, Gordon Philipp Otto, Maik Sossdorf, Christoph Sponholz, Michael Boehringer, Wolfgang Loesche, Daniel Rittirsch, Arne Wilharm, Oliver Kurzai, Michael Bauer, Ralf Alexander Claus

**Affiliations:** Center for Sepsis Control and Care (CSCC), Jena University Hospital, Erlanger Allee 101, Jena, Germany; Clinic for Anesthesiology and Intensive Care, Jena University Hospital, Erlanger Allee 101, Jena, Germany; Septomics Research Centre, Leibniz Institute for Natural Product Research and Infection Biology - Hans Knoell Institute, Beutenbergstrasse 11a, 07745 Jena, Germany; Division of Trauma Surgery, University Hospital of Zurich, Raemistraße 100, CH-8091 Zurich, Switzerland; Department of Trauma, Hand and Reconstructive Surgery, Jena University Hospital, Erlanger Allee 101, D-07743, Jena, 07747 Germany

## Abstract

**Introduction:**

Circulating histones have been identified as mediators of damage in animal models of sepsis and in patients with trauma-associated lung injury. Despite existing controversies on actual histone concentrations, clinical implications and mechanism of action in various disease conditions, histone levels in human sepsis, association with disease progression and mediated effects on endothelial and immune cells remain unreported. This study aimed to determine histone levels and its clinical implication in septic patients and to elucidate histone-mediated effects *ex-vivo*.

**Methods:**

Histone levels, endogenous activated protein C (APC) levels and clinical data from two independent cohorts of septic patients were obtained. Histone levels were compared with various control groups including healthy individuals, intensive care unit (ICU) patients without sepsis, ICU patients with multiple organ failure and patients with minor or multiple trauma, all without infection. Endothelial and monocytic cells were stimulated with histones. Cellular integrity and sepsis prototypical cytokines were evaluated. The mechanism of action of histones via Toll-like receptor 4 (TLR4) was evaluated using a function blocking antibody. Histone degradation in plasma was studied by immunoblotting.

**Results:**

Histone H4 levels were significantly elevated in patients with sepsis (cohort I; *n* = 15 and cohort II; *n* = 19) versus ICU controls (*n* = 12), patients with multiple organ failure (*n* = 12) or minor trauma (*n* = 7), associated with need for renal replacement therapy and decrease in platelet count during disease progression, and remarkably were significantly associated with increased mortality rates in septic patients (ICU-, 28 day- and 90 day mortality rates). There was an inverse correlation between plasma histones and endogenous APC levels. Histone stimulation induced the release of sepsis prototypic cytokines and decreased cell integrity indicated by a significant increase of lactate dehydrogenase (LDH) and propidium iodide (PI) staining. Blocking of TLR4 decreased cellular cytotoxicity on endothelial cells. The calculated half-life of histones in spiked plasma was 4.6 minutes.

**Conclusions:**

Histone levels in septic patients are significantly increased and might mediate disease aggravation by cellular injury and inflammation via TLR4 signaling, which potentially results in multiple organ failure and fatal outcome.

## Introduction

Sepsis is a multifactorial life-threatening syndrome arising from the immune response to invading microorganisms, resulting in excessive cell activation and tissue damage [1,2]. Based on current understanding of the pathophysiology of the host response, endothelial activation and dysfunction [3], as well as culminating multiple organ failure, are known hallmarks of the clinical course, which determines the prognosis of patients [4-6]. Multiple immune activators during systemic inflammation known as pathogen-associated molecular pattern (PAMPs) [7] and damage-associated molecular patterns (DAMPs) [8] have been reported to be involved in the aggravation of host response.

Recently reported is a concept implicating extracellular histone isoforms, mainly histone H4 (H4), as a result of host response, which aggravates maladaptive mechanisms through a direct action on endothelial cells [9] and platelets [10]. Furthermore, free histones in circulation mediate extensive cellular damage, hemostatic imbalance and amplification of the inflammatory response by inducing cytokine production as reported in an animal model of sepsis. Intravenous injection of purified histones in mice elicited thrombocytopenia, neutrophil migration, and organ failure mimicking the pathophysiological signature of sepsis [9]. Interestingly, co-infusion of histones with APC [9] or intravenous injection of histones in toll-like receptor (TLR)4-null mice abrogates histone induced effects [11]. Additionally, histones have been reported to mediate fatal liver injury in mice [11], and have recently been identified as essential effectors of C5aR- and C5L2-mediated tissue damage and inflammation in acute lung injury [12] and in trauma-associated lung injury in humans, with concentrations ranging between 10 and 280 μg/ml as measured using immunoblotting [13].

In this study, we sought to investigate histone levels in septic patients and associations with clinical data and activated protein C (APC). *In vitro*, we investigated the role of histones in cellular damage, inflammatory response and their interaction with TLR4, using functional blocking antibody.

## Materials and methods

### Clinical study

Institutional ethical approval was given by the local ethics committee of the medical faculty of Friedrich-Schiller University Hospital in Jena (2160-11/07, 2712-12/09) and the local ethics committee Zurich (*kantonale Ethikkommission Zürich*, KEK: StV26-2007), and written informed consent of each patient was obtained.

ICU controls and patients with severe sepsis or septic shock according to the American College of Chest Physicians and the Society of Critical Care Medicine (ACCP/SCCM) [14] were prospectively enrolled. Cohort I included 15 patients with sepsis from various origins during disease progression. Cohort II included 19 patients suffering from postoperative anastomosis insufficiency after major abdominal surgery (Table 1). Blood samples were collected in citrated anticoagulant tubes within 24 hours at onset of sepsis (day 1) in cohort I and at onset of sepsis, and on day 3 and day 5 in cohort II. Plasma was separated by centrifugation at 2000 × g for 10 minutes. Patient characteristics from both cohorts were obtained (Table 1). Additionally, various clinical scores and laboratory findings including age, sex, International classification of Disease 10^th^ revision (ICD-10) diagnosis, length of stay (LOS), outcome, acute physiology and chronic health evaluation II score (APACHE II), simplified acute physiology score (SAPS), sequential organ failure assessment score (SOFA), ventilator assistance, vasopressor treatment, creatinine levels and requirement for renal replacement therapy (RRT), bilirubin, C-reactive protein (CRP), partial thromboplastin time (PTT), platelet count, leukocyte count and procalcitonin levels were obtained from the patient data monitoring system. Platelet increase was defined as an increase in platelet count >30% on day 5 of sepsis progression as compared to platelet count on day 1, while platelet decrease was defined as a fall in platelet count > 30% on day 5 of sepsis as compared to platelet count at onset of sepsis.

**Table 1:** **Clinical characteristics of patients at study enrollment**

**Patient characteristics**	**Cohort I**	**Cohort II**
Number of patients (n)	15	19
Age, years, median (IQR)	72 (48 to 77)	69 (45 to 73)
Gender male, n (%)	8 (53.3)	13 (68.4)
ICU survivor, n (%)	10 (66.7)	12 (63.2)
28-day survivors, n (%)	n.d.	14 (73.7)
90-day survivors, n (%)	n.d.	7 (36.8)
APACHE-II, median (IQR)	24 (18 to 30)	19 (15 to 26)
SAPS-II, median (IQR)	44 (33 to 73)	44 (34 to 52)
SOFA, median (IQR)	10 (6 to 13)	10 (6 to 11)

APACHE II, acute physiology and chronic health evaluation II; SAPS, simplified acute physiology score; SOFA, sequential organ failure assessment score.

n.d. not determined.

For comparison of histone concentrations, we included various control groups, which included; 5 healthy individuals, 12 ICU patients without sepsis, 12 ICU patients with multiple organ failure (MOF), 7 patients with distal radius fracture (minor trauma), and 12 patients with multiple trauma (nonconsecutive patients with thoracic injury; age: median 73 years, range 42 to 92 years; injury severity score (ISS): median 34, range 17 to 66; with blunt chest trauma; male/female: 8/3; lethality 27.3% [3/11]). The control groups showed no signs of acute infection. Collection of trauma samples was performed following completion of other groups.

### Enzyme-linked immunosorbent assay (ELISA)

Human histone H4 and APC ELISA kits were obtained from USCN Life Science (Wuhan, China). Standards and patient samples were run in duplicates according to manufacturer’s instructions.

### Stimulation assays

Commercially available calf thymus histones (Sigma, Deisenhofen, Germany) were passed through a high affinity endotoxin detoxi-gel (Thermo Fisher Scientific, Waltham, Massachusetts, USA) before use in all stimulation assays to remove potential endotoxin contamination. Purified calf thymus histones were used for stimulatory experiments on immortalized human microvascular endothelial cells (HMEC) and human monocytic cells (MM6). For *in vitro* assays, 500,000 cells per well seeded in six-well plates were stimulated with 10 ng/ml and 50 μg/ml of histones in cell culture media supplemented with 1% fecal calf serum. Unstimulated cells were used as negative control. Experiments were performed at least in triplicates.

### Human TLR4 neutralization

Functional antibody against human TLR4 (PAb hTLR4) and control antibody (PAb Control) were obtained from Invivogen (San Diego, CA, USA). A total of 25,000 cells were seeded in 96-well plates. Antibodies were diluted to a final concentration of 5 μg/ml and incubated at 37°C for 10 minutes. A 50 μg/ml histone concentration was used for stimulation and cells were incubated overnight for 24 hours. Supernatant was collected for lactate dehydrogenase (LDH) measurements and cells were stained with propidium iodide (PI).

### Fluorescent staining (Propidium iodide staining)

Cells were detached with 1X trypsin and washed three times with 1X sterile PBS. Cells were resuspended in 1 ml of 1X PBS and incubated with 10 μg/ml PI dye solution (Sigma, St. Louis, USA) in the dark for 5 minutes at room temperature. Fluorescent intensity was measured by flow cytometry.

### Lactate dehydrogenase measurement

LDH levels in cell culture supernatant were measured at 0 hours and 24 hours after histone stimulation with a commercially available kit (Roche, Germany) according to manufacturer’s instructions. Absorbance was read at 490 nm using a spectrophotometer.

### Cytokine measurements

For quantification of cytokines in cell culture supernatant after histone stimulation, a cytometric bead assay (CBA) was performed according to the manufacturer’s instructions (human inflammation kit; BD Biosciences, Germany) and measured by flow cytometry using a FACS calibur.

### Determination of histone stability

Blood from three healthy volunteers was drawn into citrated anticoagulant tubes and plasma was separated by centrifugation at 2000 × g for 10 minutes. Plasma was spiked with calf thymus histones to a concentration of 100 μg/ml and incubated at 37°C with mild shaking for 5, 10, 15 and 30 minutes. Plasma was separated by western blotting and detection of histones was performed using anti-histone H3 antibodies (Cell signalling, USA). Determination of half-life was performed by approximation of the degradation process reaching a plateau phase.

### Statistical analysis

Levels of histone measurements are given as median including the 25^th^ and 75^th^ IQR. Analysis of variance (ANOVA) on ranks was used to determine differences between histone concentrations at onset of sepsis, day 3 and day 5. The Student *t*-test or Mann-Whitney test was used to compare independent variables where applicable. Multivariate linear regression analysis was used to predict clinical characteristics that were independently associated with histone levels. Spearman correlation coefficient (*r*) was used to determine the correlations between independent parameters and histone levels. Statistical significance was set at *P* <0.05. Densitometry analysis was performed using AIDA software and a single-phase decay analysis for calculation of half-life was performed using Graph Pad Prism 5.0.

## Results

### Histone levels in septic patients correlate with disease progression and mortality

**Figure 1:**
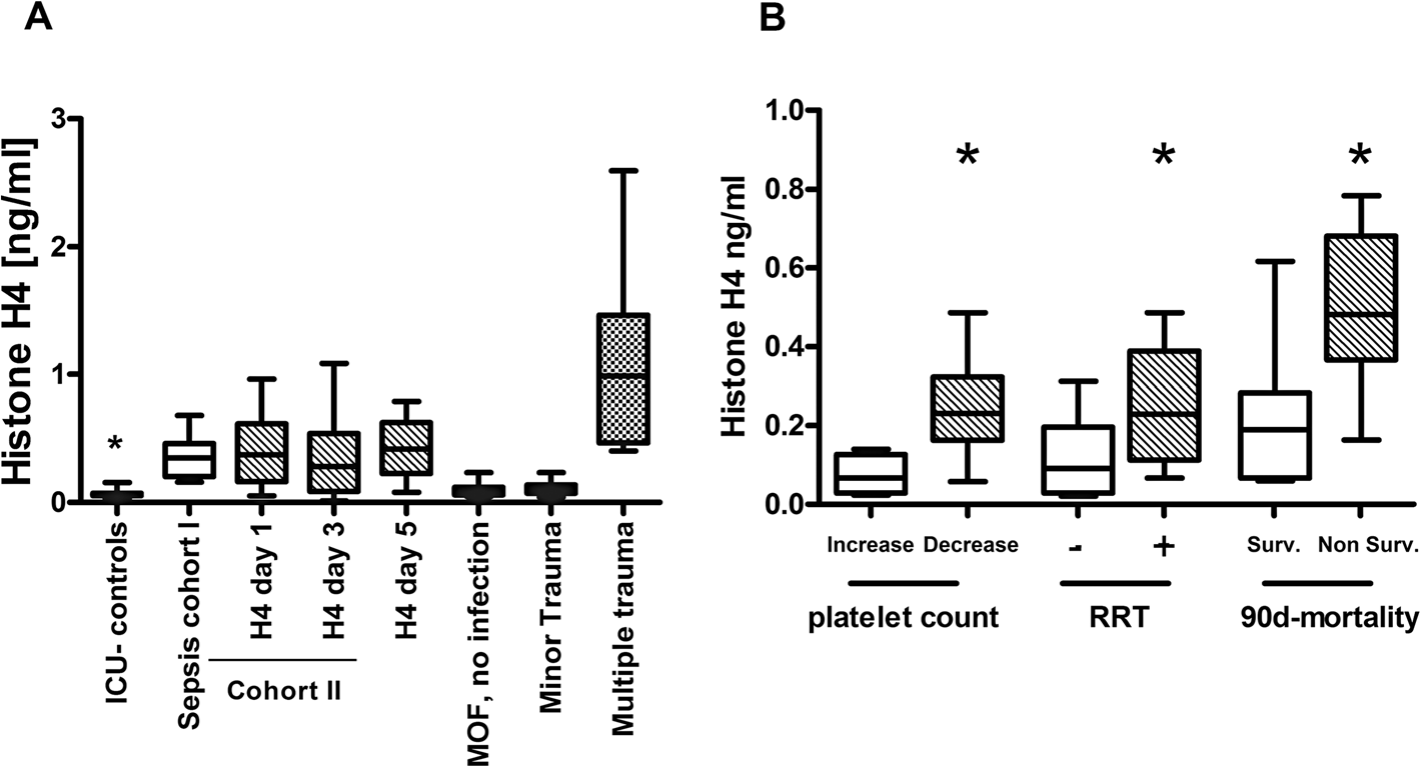
**Plasma histone H4 concentration in two independent cohorts of septic patients (Cohorts I and II) and associations with disease progression and mortality. (A)** Plasma histone concentration in sepsis cohort I (n = 15) and sepsis cohort II (n = 19) on day 1, day 3, day 5, ICU controls (n = 12), patients with multiple organ failure (MOF) (n = 12), minor trauma (n = 7) and multiple trauma (n = 12) patients. All controls were without signs of infection. *Mann-Whitney test (*P* <0.05) to compare independent groups. **(B)** Plasma histone H4 at day 1 associated with platelet count at day 5, requirement for renal replacement at day 5, and outcome at day 90. *Mann-Whitney test, *P* <0.05. RRT^**+**^ indicates the requirement of renal replacement therapy; RRT^**−**^ indicates no requirement for renal replacement therapy.

In cohort I, histone H4 levels were significantly elevated compared to ICU controls (sepsis cohort I: median 0.35, IQR 0.2 to 0.46) versus ICU controls: median 0.06 (0.05 to 0.07) ng/ml, *P* <0.05; Figure 1A). In cohort II, histone H4 levels were significantly elevated during the course of sepsis on day 1, day 3 and day 5 as compared to the ICU control group (sepsis cohort II, day 1: median 0.37 (0.16 to 0.61), day 3: median 0.28 (0.08 to 0.53), day 5: median 0.41 (0.22 to 0.62) versus ICU controls: median 0.06 (0.05 to 0.07) ng/ml, *P* <0.05; Figure 1A). Histone concentrations in both cohorts ranged from 0.01 to 1.08 ng/ml with an inter-assay coefficient of variation (CV) <10%. However, detection of histones in plasma of patients by immunoblotting was not possible because the observed concentrations were far below the limit of detection by this method (approximately 500 ng/ml). Histone levels on day 1 in both cohorts of septic patients were also significantly elevated compared to patients with MOF (sepsis cohort I: median 0.35 (IQR 0.2 to 0.46), sepsis cohort II: median 0.37 (0.16 to 0.62) versus MOF: median 0.08 (IQR 0.05 to 0.11) ng/ml, *P* <0.05) and minor trauma patients (sepsis cohort I: median 0.35 (IQR 0.2 to 0.46), sepsis cohort II: median 0.37 (0.16 to 0.62) versus minor trauma: median 0.11 (IQR 0.07 to 0.13) ng/ml, *P* <0.05). However, histone levels in multiple trauma patients were 3-fold higher than levels measured in septic patients with concentrations up to 3 ng/ml (multiple trauma: median 0.98 (IQR 0.46 to 1.46) versus sepsis cohort I: median 0.35 (IQR 0.2 to 0.46), sepsis cohort II: median 0.37 (0.16 to 0.62) ng/ml, *P* <0.05, Figure 1A).

Analyses of the clinical data from cohort II revealed that patients with a decline in platelet count >30% on day 5 of sepsis progression demonstrated significantly elevated histone levels on day 1 compared to patients with an increase in platelet count >30% (decreased platelets: median 0.23 (0.16 to 0.32) versus increased platelets: median 0.06 (0.02 to 0.12) ng/ml, *P* <0.05; Figure 1B). Due to unvaried platelet count over time, two patients were not included in this subgroup analysis. Furthermore, patients who required RRT until day 5 presented significantly elevated histone levels on day 1 as compared to patients without subsequent need for RRT (need for RRT: median 0.23 (0.11 to 0.38) versus without need for RRT: median 0.09 (0.02 to 0.19) ng/ml, *P* <0.05; Figure 1B). With respect to ICU- and 28-day mortality, on day 1 non-survivors presented with significantly elevated concentrations compared to survivors (ICU mortality, non survivors: median 0.54 (0.34 to 0.71) versus survivors: median 0.21 (0.06 to 0.4) ng/ml, *P* <0.05; 28-day mortality, non survivors: median 0.67 (0.25 to 0.75) versus survivors: median 0.25 (0.07 to 0.45) ng/ml, *P* <0.05). Remarkably, this was also evident for 90-day mortality (non-survivors: median 0.48 (0.36 to 0.68) versus survivors: median 0.19 (0.06 to 0.28) ng/ml, *P* <0.05; Figure 1B). Multivariate linear regression analysis including APACHE-II score, SOFA score, CRP, white blood cells (WBC), creatinine, and platelet count as independent variables identified WBC (*P* <0.05, *R*^2^ = 0.64) and SOFA score (*P* <0.05, *R*^2^ = 0.64) as predictors for histone levels at onset of sepsis (day 1).

### Histone levels negatively correlate with endogenous APC

**Figure 2:**
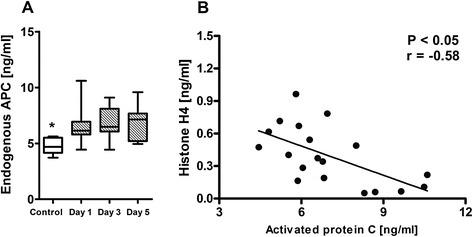
**Endogenous APC levels in patients with sepsis and correlation with plasma histone levels. (A)** Endogenous activated protein C (APC) levels in patients at onset of sepsis (day 1), day 3 and day 5. *Analysis of variance on ranks test (*P* <0.05) to compare difference between APC levels on day 1, day 3 and day 5. **(B)** Endogenous APC levels negatively correlated with plasma histone levels. *Spearman correlation (*P* <0.05).

Endogenous APC levels in cohort II at day 1, day 3 and day 5 were analyzed. APC levels were significantly elevated as compared to healthy controls (day 1: median 6.14 (IQR 5.8 to 6.9), day 3: median 6.49 (6.06 to 8.10), day 5: median 7.15 (5.21 to 7.68) ng/ml versus controls: median 4.69 (4.16 to 5.51) ng/ml; *P* <0.05; Figure 2A). APC levels were inversely correlated with histone levels (*r* = −0.584, *P* <0.05; Figure 2B). Furthermore, elevated APC levels were identified as an independent predictor of decreased histone levels when included in the multivariate linear regression model (*P* <0.001, *R*^2^ = 0.723).

### Histones mediate cellular damage and induced the cytokines production

**Figure 3:**
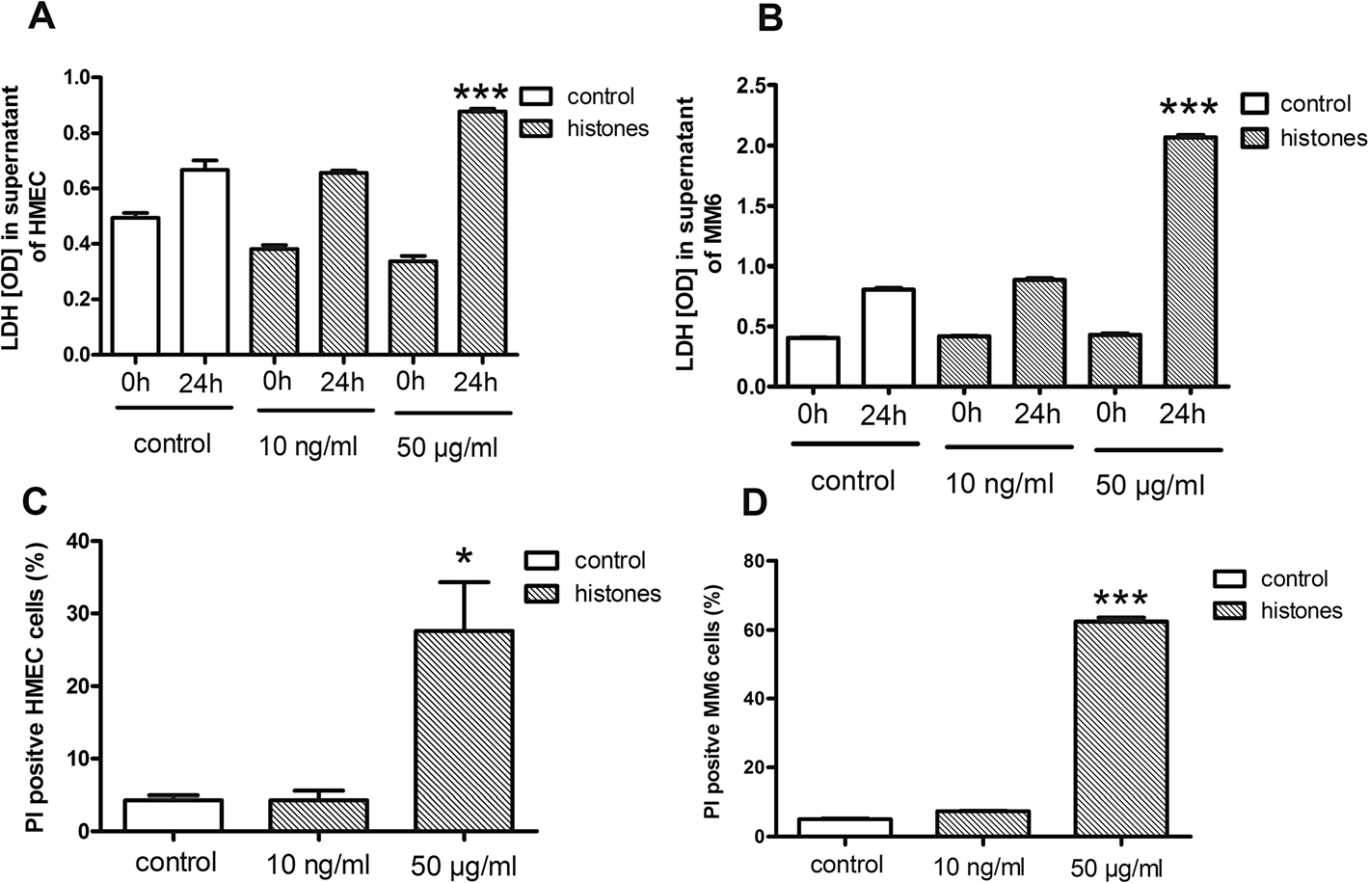
**Evaluation of cellular integrity and viability after histone stimulation of endothelial and monocytic cells. (A)** Lactate dehydrogenase (LDH) levels significantly increased in supernatant of human microvascular endothelial cells (HMEC) after 50 μg/ml histones stimulation for 24 h. ***Student’s *t*-test, *P* <0.001. **(B)** LDH levels significantly increased in supernatant of mono mac 6 (MM6) cells after 50 μg/ml histones stimulation for 24 h. ***Student’s *t*-test, *P* <0.001. **(C)** Percentage of propidium iodide (PI)-positive HMEC cells significantly increased after 24 hours stimulation with 50 μg/ml histones. *Student’s *t*-test, *P* <0.05. **(D)** Percentage of PI-positive MM6 cells significantly increased after 24 hours stimulation with 50 μg/ml histones. ***Student’s *t*-test, *P* <0.001.

**Figure 4:**
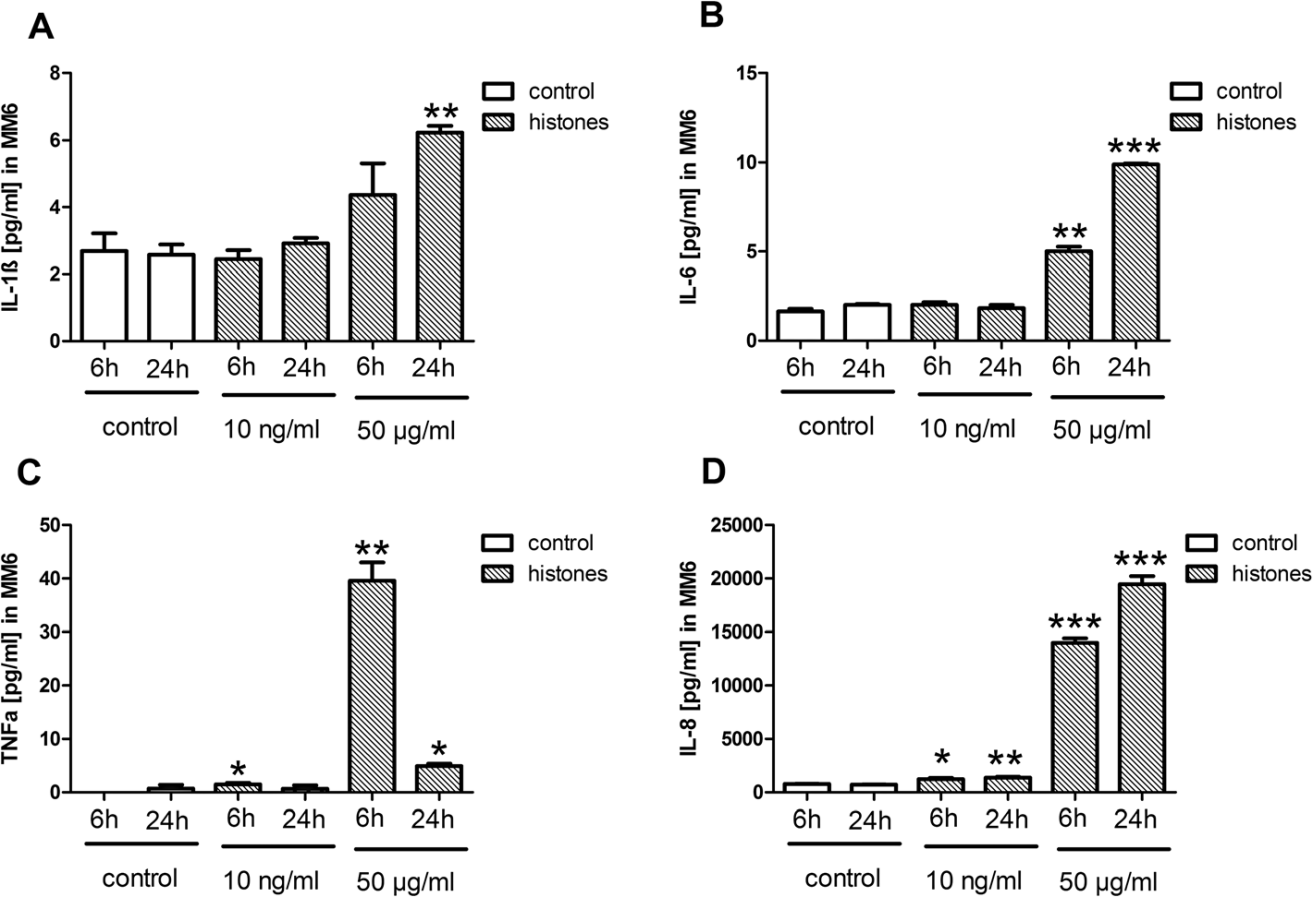
**Cytokine production after histone stimulation. (A)** IL-8 levels in supernatant from mono mac 6 (MM6) significantly increased after 10 ng/ml (*P* < 0.05) and 50 μg/ml stimulation for 6 and 24 hours. ***Student’s *t*-test, *P* <0.001. **(B)** IL-1ß levels in supernatant from MM6 significantly increased after 50 μg/ml stimulation for 24 hours. **Student’s *t*-test, *P* <0.05. **(C)** IL-6 levels in supernatant from MM6 significantly increased after 50 μg/ml stimulation for 6 and 24 hours. ***Student’s *t*-test, *P* <0.001. **(D)** TNF-α levels in supernatant from MM6 increased after 6 hours with 10 ng/ml and 50 μg/ml histone stimulation and 50 μg/ml stimulation after 24 hours. *Student’s *t*-test, *P* <0.05.

The cytotoxic effects of histones on endothelial cells and monocytes using 10 ng/ml and 50 μg/ml histone concentration were evaluated. There was no change in LDH release or PI fluorescence of the nuclei after stimulation with 10 ng/ml. Histone concentration of 50 μg/ml led to cell death indicated by significant high levels of LDH and PI positive cells (*P* <0.001) (Figure 3A-D). The stimulation of monocytes with 10 ng/ml histone concentration led to a significant increase in TNF-α at 6 hours and IL-8 (6 hours, 24 hours, *P* <0.05) while 50 μg/ml histones led to a marked increase in sepsis-associated cytokines such as TNF-α, IL-6, IL-8, and IL-1ß at 6 hours and IL-6, IL-8, and IL-1ß at 24 hours (Figure 4A-D).

### Blocking anti-TLR4 antibodies inhibits histone-induced cytotoxicity

**Figure 5:**
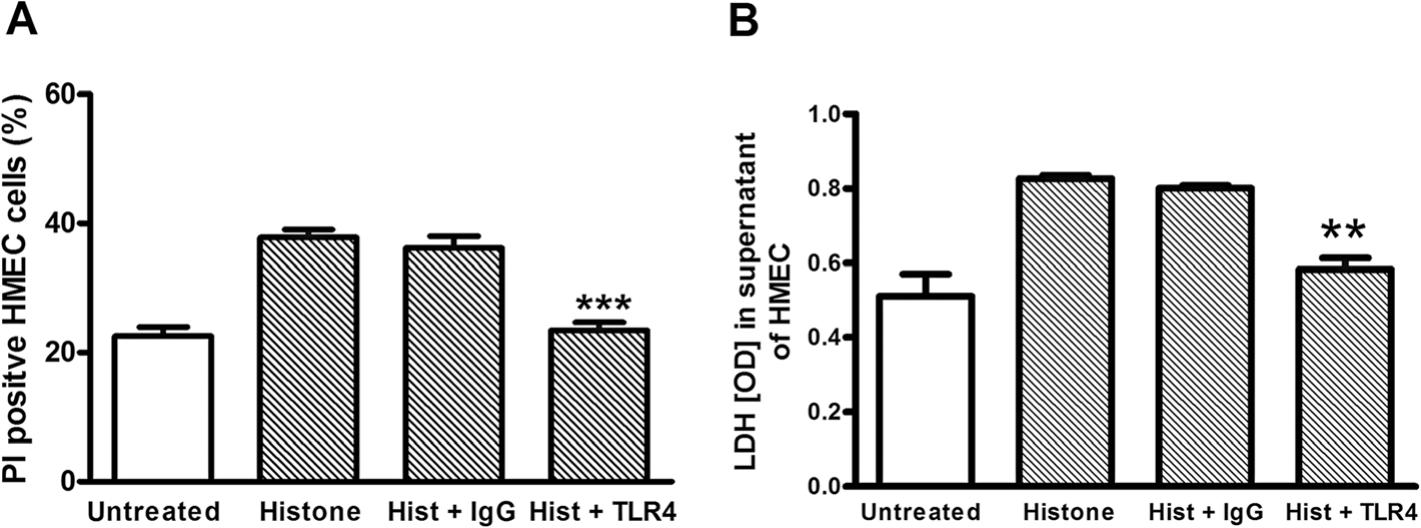
**Histone effects after toll-like receptor 4 (TLR4) blockade.** Blocking antibody control was obtained from animals housed under the same condition as immunized animals to produce TLR4 antibody. **(A)** Percentage of propidium iodide (PI)-positive human microvascular endothelial cells (HMEC) after 24 hours stimulation with 50 μg/ml histones with and without blocking of TLR4. ***Student’s *t*-test, *P* <0.001. **(B)** Lactate dehydrogenase (LDH) release significantly decreased after blocking with specific TLR4 antibody compared to histone-stimulated cells. **Student’s *t*-test, *P* <0.001.

To evaluate the mode of action of histones, TLR4 signaling via receptor neutralization was targeted. Pre-treatment of endothelial cells with a specific TLR4 blocking antibody abrogated histone-induced increase of cellular cytotoxicity as shown by both PI staining and measurement of LDH release (*P* <0.05) after stimulation with 50 μg/ml histones (Figure 5A-B).

### Determination of histone stability

**Figure 6:**
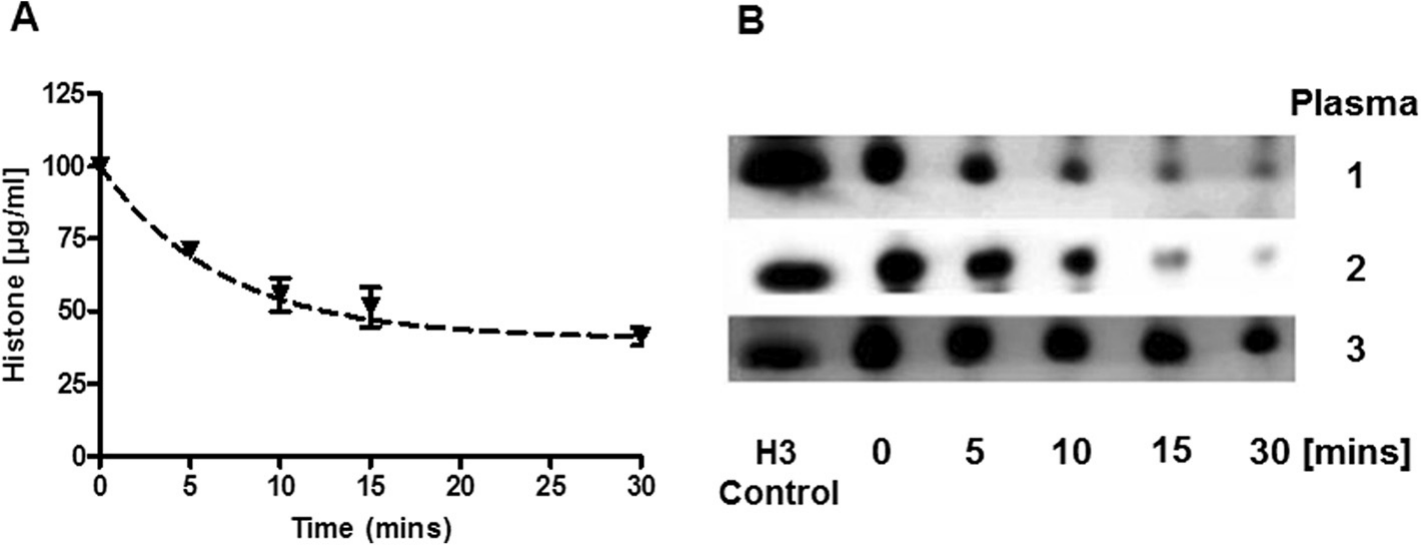
**Estimated degradation kinetics of histones in plasma. (A)** Single-phase exponential decay fit of three healthy individuals and the margin of error between samples. The half-life of histones (k) was 4.6 minutes with an *R* ^2^ = 0.9159. **(B)** Western blotting showed decrease in histone protein in plasma over time.

In histone-spiked plasma, histone degradation over time was measured by western blotting. We found a single nonlinear regression exponential decay of histones in plasma between 0 to 30 minutes. The half-life of histones in three independent plasma samples was 4.6 minutes with an *R*^2^ of 0.9159 (Figure 6A-B). Interestingly, after 30 minutes, degradation reached a plateau and no further decline was observed up to 6 hours (data not shown).

## Discussion

The study demonstrated for the first time and in two independent cohorts of septic patients that plasma concentrations of extracellular histones are elevated during human sepsis as compared to ICU controls. Interestingly, the observed concentrations are similar in both cohorts with low inter-assay variation. Histones were also significantly increased during the course of disease progression until day 5 of sepsis. This is in line with previous reports of an increase in histone concentration in baboons after endotoxin challenge [9]. Interestingly, histone levels were higher in septic patients compared to patients with multiple organ failure without infection, potentially indicating that the source of histones in circulation could be strongly associated with the underlying infection and the release of neutrophil extracellular traps by neutrophils as demonstrated by others [15,16]. In addition, we found lower levels of histones in patients with minor trauma. This might be related to the limited tissue damage representing a lower release of histones into circulation, possible clearance of small quantities of histones in circulation by macrophages or complete eradication through active cleavage by proteases in plasma such as endogenous activated protein C. Most interestingly, we observed that in our cohort of patients with multiple trauma, histone concentrations were higher than levels found in septic patients. This supports previous reports of elevated levels of histones found in patients with blunt traumatic lung injury. Nevertheless, there was a clear disparity in the concentrations of histones measured in our multiple trauma patient cohort in the nanogram range compared to the levels reported in traumatic lung injury in the microgram range [13]. However, we could not confirm histone concentrations by mass spectrometry due to high abundance plasma proteins, which interfere with the detection of low abundance proteins despite the existence of high abundance protein depletion protocols. Our efforts to quantify histones by immunoblotting also failed because levels of histones found in patients were far below the lower limit of detection for immunoblotting (>500 ng/ml). Nevertheless, our investigation using two independent cohorts of sepsis patients demonstrates and justifies consistency and reproducibility of plasma histone measurements in septic patients. In addition, the inclusion of a second cohort enabled us to determine plasma histones not only at onset of sepsis but also during disease progression.

A hallmark of sepsis is the occurrence of renal failure requiring renal replacement therapy [17]. Consistently, our data suggested that higher levels of histones correlated with the need for RRT. In addition, histones have been shown to bind to platelets recruiting plasma adhesion molecules, thereby promoting platelet aggregation [10]. In our study, increased histone levels were associated with a decrease in platelet count corroborating this concept. Interestingly, the occurrence of renal failure in sepsis and thrombocytopenia is often associated with high mortality [18,19]. The observed increase of plasma histone levels in patients undergoing RRT also implies that this increase might be a consequence of impaired renal excretion of the protein. However, to the best of our knowledge, there is no evidence that histones are excreted and become present in urine, either in healthy or pathophysiological conditions. On the other hand, microvascular permeability is increased when renal microvasculature is exposed to extracellular histones, released from dying tubular epithelial cells [20]. In lipopolysaccharide (LPS)-induced endotoxemia, neutralization of released histones reduced tubular injury and an improved renal function as measured by creatinine levels was obvious [20], identifying histones as mediators of acute kidney injury. Strikingly, higher histone levels correlated with ICU-, 28- and 90-day mortality. Interestingly, thrombocytopenia and organ dysfunction following histone administration have also been demonstrated in mice [8,10]. Based on this concept, it could be hypothesized that the interaction of histones with the endothelium will result in endothelial impairment. This damage leads to endothelial dysfunction promoting platelet activation and aggregation, which impairs microcirculation and results in organ dysfunction, which is associated with an increased risk of death [21]. In line with this, our *ex-vivo* findings further demonstrate that histone mediated cytotoxicity on endothelium cells and underline the results of Fuchs *et al*. demonstrating direct platelet activation and aggregation [10]. Besides the direct effects on platelets and cytotoxicity, we were able to show an activation of immune cells which led to an increase in sepsis prototypic cytokines in both low and high concentrations of histone stimulation. From a pathomechanistic perspective, histone mediated effects might be initiated by TLR4 signaling as neutralization or blocking of the TLR4 receptor in our study completely abrogated histone-mediated cytotoxicity in our *in vitro* model. This underlines a report by Xu *et al*. who showed that TLR4 knock-out mice are protected from the fatal effects of histone infusion [11] and also, at least in part, is in line with results from Abrams *et al*. showing that TLR4 blocking results in a decrease in cytokine production but not cytotoxicity.

We report lower concentrations of histones in the nanogram range during sepsis which contradicts reports on the levels of histones measured in various species and diseases (baboons after gram-negative challenge, approximately 15 μg/ml [9], in patients with blunt traumatic lung injury, approximately 10 to 280 μg/ml [13], 200 μg/ml in mice models of acute lung injury [12]) and reported concentration used in all histone stimulation studies so far (approximately 10 to 1000 μg/ml) [9,11-13,22]. It could also be speculated that the discrepancy in histone levels released in animal (mice and baboons) sepsis and the actual concentration measured in plasma of our septic patients might be due to the treatment with heparin in our septic ICU patients. Heparin is highly negatively charged and was recently shown to bind to positively charged histones reducing their cytotoxic effect [22]. In line with other reports, the observed histone levels in multiple trauma patients might be related to a higher degree of tissue damage as represented by the injury severity score [13]. In our cohort stratified according to need for RRT on day 5 (cohort II), all of the patients underwent anticoagulation either with heparin (2/3 of all our ICU patients) or citrate (1/3 of all our ICU patients). However, measurements of histones to associate the level with the clinical endpoint of need for RRT were performed prior to initiation of anticoagulation for supportive treatment. From the low number of samples with different anticoagulants, from which one might interact with the readout, no comparison between these was performed.

We demonstrate also for the first time the ability of plasma to degrade histones. As plasma was used for these experiments, a potential binding of positively charged histones to negatively charged membranes of erythrocytes could be excluded. The observed fast initial degradation of histones might be related to free proteases within the plasma such as endogenous APC and might explain why co-injection of histones with APC abrogated the lethal effects of histone injection in mice [9]. This might also support our finding of a negative correlation between histone levels and APC in our septic patients. Unclear, however, is the decline to a steady state of histones in plasma after 30 minutes. However, we speculate that the biological half-life of histones in plasma could be affected by active metabolites, binding of fragments to other proteins as well as receptor interactions and therefore will not follow the first order kinetics with a fixed rate constant, because the generated fragments were also detected by the cited polyclonal antibodies. Nevertheless, this finding indicates the need for early centrifugation and measuring or freezing of the samples. Finally, further studies should elucidate the origin and sources of circulating histones in plasma during sepsis.

## Conclusions

In conclusion, our data provides evidence that plasma histones are not only elevated in human sepsis but are associated with sepsis-related organ dysfunction, mediating cellular damage and severe inflammation responses. The observed effects might be mediated via the TLR4 receptor.

## Key messages

- We analyzed histone concentrations in two independent cohorts of septic patients. Histone levels were significantly elevated in septic patients as compared to ICU controls but lower in patients with multiple trauma.
- Higher concentrations of histones associated significantly with lower endogenous APC levels, with the requirement for RRT, a decrease in platelet count and were found to be higher in 28- and 90-day non-survivors.
- *In vitro*, histone stimulation of cultured endothelial and monocytic cells decreased cellular integrity and resulted in an increase in septic prototypic cytokines.
- Histone-mediated action might be through TLR4. The half-life of histone degradation in plasma was 4.6 minutes.

## Abbreviations

ACCP: American College of Chest Physicians
ANOVA: analysis of variance
APACHE: II acute physiology and chronic health evaluation II
APC: activated protein C
CBA: cytometric bead array
CRP: C-reactive protein
CV: coefficient of variation
DAMP: damage-associated molecular pattern
ELISA: enzyme linked immunosorbent assay
H4: histone 4
HMEC: human microvascular endothelial cells
ICD-10: International Classification of Disease 10th revision
IL: interleukin
LDH: lactate dehydrogenase
LOS: length of stay
MM6: mono mac 6
MOF: multiple organ failure
PAMP: pathogen-associated molecular pattern
PBS: phosphate buffered saline
PI: propidium iodide
PTT: partial thromboplastin time
RRT: renal replacement therapy
SAPS: simplified acute physiology score
SCCM: Society of Critical Care
SOFA: sequential organ failure assessment score
TLR4: toll-like receptor 4
TNF: tumor necrosis factor
WBC: white blood cells
